# Sequencing and Analysis of the Complete Organellar Genomes of *Prototheca wickerhamii*

**DOI:** 10.3389/fpls.2020.01296

**Published:** 2020-09-01

**Authors:** Zofia Bakuła, Robert Gromadka, Jan Gawor, Paweł Siedlecki, Jan J. Pomorski, Kacper Maciszewski, Agnieszka Gromadka, Anna Karnkowska, Tomasz Jagielski

**Affiliations:** ^1^ Department of Medical Microbiology, Faculty of Biology, Institute of Microbiology, University of Warsaw, Warsaw, Poland; ^2^ DNA Sequencing and Oligonucleotides Synthesis Laboratory at the Institute of Biochemistry and Biophysics, Polish Academy of Sciences, Warsaw, Poland; ^3^ Department of Systems Biology, University of Warsaw, Warsaw, Poland; ^4^ Institute of Biochemistry and Biophysics, Polish Academy of Sciences (PAS), Warsaw, Poland; ^5^ Museum and Institute of Zoology, Polish Academy of Sciences, Warsaw, Poland; ^6^ Institute of Evolutionary Biology, Faculty of Biology, Biological and Chemical Research Centre, University of Warsaw, Poland

**Keywords:** *Prototheca wickerhamii*, protothecosis, colorless algae, mitochondrial genome, plastid genome

## Abstract

Of the *Prototheca* genus, *Prototheca* *wickerhamii* has the highest clinical significance in humans. However, neither nuclear nor organellar genomes of this species were sequenced until now. The hitherto determined and analyzed mitochondrial and plastid genomes of the alleged *P. wickerhamii* species belong in fact to another species, recently named *Prototheca xanthoria*e. This study provides a first insight into the organellar genomes of a true *P. wickerhamii* (type strain ATCC 16529). The *P. wickerhamii* mitochondrion had a 53.8-kb genome, which was considerably larger than that of *Prototheca* *ciferrii* (formerly *Prototheca zopfii* gen. 1) and *Prototheca bovis* (formerly *Prototheca zopfii* gen. 2), yet similarly functional, with the differences in size attributable to a higher number of introns and the presence of extra unique putative genes. The 48-kb plastid genome of *P. wickerhamii*, compared to autotrophic Trebouxiophyceae, was highly reduced due to the elimination of the photosynthesis-related genes. The gene content of the plastid genome of *P. wickerhamii* was, however, very similar to other colorless *Prototheca* species. Plastid genome-based phylogeny reinforced the polyphyly of the genus *Prototheca*, with *Helicosporidium* and *Auxenochlorella* branching within clades of *Prototheca* species. Phylogenetic reconstruction also confirmed the close relationship of *P. wickerhamii* and *P. xanthoriae*, which is reflected in the synteny of their organellar genomes. Interestingly, the entire set of *atp* genes was lost in *P. wickerhamii* plastid genome while being preserved in *P. xanthoriae*.

## Introduction

The genus *Prototheca* comprises unicellular, nonphotosynthetic, saprophytic microalgae, usually associated with humid and organic-rich environments. These organisms are unique in the Plantae kingdom in that they have consistently been implicated in human and animal infections, collectively referred to as protothecosis (Jagielski and Lagneau, 2007). The taxonomy of the *Prototheca* genus has recently been revised based on phylogenetic analysis of partial *cytb* gene sequences. In the light of this new classification, 14 *Prototheca* species are proposed, split into two major lineages, comprising either human- or cattle-associated species (Jagielski et al., 2019).

The genus *Prototheca* along with another nonphotosynthetic genus *Helicosporidium* belongs to the predominantly photosynthetic clade of Trebouxiophyceae green algae. All *Prototheca* species evolved from photosynthetic algae that had lost their ability to photosynthesize yet retained vestigial plastids with substantially reduced genome (Suzuki et al., 2018). It has been suggested that the loss of photosynthesis happened independently three times in this lineage (Suzuki et al., 2018), and that might be reflected in the gene order and gene complement of the vestigial plastid genomes.

*Prototheca wickerhamii* represents the predominant etiological agent of human protothecosis, affecting both immunocompetent and immunocompromised patients (Todd et al., 2018). Clinically, the disease most frequently involves the skin and subcutaneous tissue followed by articular and disseminated manifestations. Treatment of protothecal infections is often difficult due to resistance of the algae to multiple antimicrobial agents (Lass-Flörl and Mayr, 2007; Todd et al., 2018).

Despite the pathogenic potential of *Prototheca* spp., the scientific knowledge on this genus remains very limited. Even more scarce are the genetic-level data so far accumulated for the *Prototheca* algae. Genome-wide sequencing has been attempted only thrice for four *Prototheca* species, namely *Prototheca ciferrii* (formerly *P. zopfii* gen. 1; strains: SAG 2063; 18125; N71), *Prototheca bovis* (formerly *P. zopfii* gen. 2; strains: SAG 2021; 50779), *Prototheca cutis* (JCM 15793), and *Prototheca* *stagnora* (JCM 9641) (Severgnini et al., 2018; Suzuki et al., 2018; Zeng et al., 2019). Furthermore, the previously reported mitochondrial (Wolff et al., 1994; Wolff and Kück, 1996) and plastid (Yan et al., 2015) genomes of *P. wickerhamii* were from the alleged *P. wickerhamii* strain SAG 263-11, which, according to the current taxonomy, represents a different species—*Prototheca xanthoriae* (Jagielski et al., 2019). Overall, studies at the genetic level may disclose the acquisition and evolution of the pathogenicity in *Prototheca* spp. as well as species-specific differences in the infectivity, pathogenicity, and clinical course (severity) of *Prototheca* infections.

In this work, we report, for the first time, the complete organellar genomes of the true *P. wickerhamii* species.

## Materials and Methods

The type strain of *P. wickerhamii* (ATCC 16529) was used in the study. Genomic DNA was extracted with a previously described protocol (Jagielski et al., 2017).

Whole genome sequencing (WGS) was performed with a combination of the Illumina MiSeq (Illumina, USA; paired-end, 2 × 300 bp) and PacBio (Pacific Biosciences, USA) platforms using the manufacturer’s standard protocols.

Sequencing of the organellar *P. wickerhamii* DNA yielded a total of 134,966 and 9,601 reads for Illumina and PacBio respectively. This accounted for 1.1% (Illumina, USA) and 3.3% (PacBio) of the total number of reads for the entire *P. wickerhamii* genome.

Sequence reads were quality filtered and trimmed using FASTX toolkit (Pearson et al., 1997) and Cutadapt (Martin, 2011), respectively. The PacBio read sets were assembled *de novo* using wtdbg2 software (Ruan and Heng, 2019) and then corrected using Illumina data with Pilon software (Walker et al., 2014). All bioinformatics manipulations were done using the SeqMan software (DNAStar, USA) and CLCBio Genomic Workbench NGS pipeline (CLCBio, Denmark).

RNA was isolated using Total RNA kit (A&A Biotechnology, Poland) with RNase-free DNase (A&A Biotechnology, Poland) treatment step. Libraries were generated and sequenced according to the producer’s protocol on a MiSeq instrument (Illumina, USA).

Gene prediction and annotation of organellar DNA were performed using the GeSeq ver. 1.76 (Tillich et al., 2017). To predict tRNA genes, online tools tRNAscan v2.0.3 (Chan and Lowe, 2019), MFannot (https://megasun.bch.umontreal.ca/cgi-bin/dev_mfa/mfannotInterface.pl) and RNAweasel (https://megasun.bch.umontreal.ca/cgi-bin/RNAweasel/RNAweaselInterface.pl) were used. Automatically generated gene models have been validated manually using Artemis v.18.0.3 genome browser (Carver et al., 2012). Additionally, localization of rRNA genes was confirmed by manually comparing with RNAseq data. Mitochondrial and plastid genomes of *P. wickerhamii* were compared with the reference genomes listed in **Table 1**, which had been annotated previously (Severgnini et al., 2018). Apart from the *Prototheca* spp., the comparative analysis included other Trebouxiophyceae green algae, *Chlorella variabilis*, and the closest *Prototheca* relatives, *i.e.* the photosynthetic, mixotrophic alga *Auxenochlorella protothecoides*, and nonphotosynthetic obligate entomoparasite *Helicosporidium* sp.

**Table 1:** General features of the mitochondrial and plastid genomes of *Prototheca* spp., and closely related species, i.e. *C. variabilis*, *A. protothecoides*, and *Helicosporidium* sp.

	Species	Size (bp)	No. of scaffolds	%GC	Total features^a^	CDS^b^	tRNA	rRNA	No. of introns (size; bp)	GenBank Acc. No.
Mitochondrion	*P. wickerhamii*	53,822	1	25.81	67	38 (5)	27(+1)^e^	3	5 (7,016)	MN794237
	*P. xanthoriae* ^c^	55,328	1	25.8	65	36 (7)	26	3	5 (4,709)	NC_001613.1
	*P. ciferrii*	38,164	1	28.7	62	33	26	3	0 (0)	MF197533.1
	*P. bovis*	39,222	1	28.7	63	35	26	3	1 (776)	MF197534.1
	*Helicosporidium* sp.	49,343	1	25.6	65	37 (5)	25	3	2 (8,208)	NC_017841.1
	*A. protothecoides*	57,274	1	28.7	68 (+2)^d^	39 (6)	26	3	7 (6,589)	NC_026009.1
	*C. variabilis*	78,500	1	28.2	62	32	27	3	6 (5,482)	NC_025413.1
Plastid	*P. wickerhamii*	47,997	1	28.2	67	35	30(+1)^e^	3	4 (561)	MN794236
	*P. xanthoriae* ^c^	55,636	1	31.1	70	40	27	3	0 (0)	KJ001761.1
	*P. cutis*	51,673	1	29.7	72	40	29	3	0 (0)	AP018373.1
	*P. stagnora*	48,188	1	25.7		28 (4)	25	3	0 (0)	AP018372.1
	*P. ciferrii*	28,698	1	27.0	47	19	25	3	0 (0)	MF197535.1
	*P. bovis*	28,638	1	26.8	47	19	25	3	0 (0)	MF197536.1
	*Helicosporidium* sp.	37,454	1	26.9	54	26	25	3	1 (486)	NC_008100.1
	*A. protothecoides*	84,576	1	30.8	109	76	30	3	0 (0)	NC_023775.1
	*C. variabilis*	124,793	1	34.0	112	79	30	3	3 (1,657)	NC_015359.1

An updated **Table 1** from Severgnini et al. (2018).

a Total no. of features include CDS (Coding DNA Sequence), tRNA and rRNA.

b CDS. No. of ORFs not previously characterized (hypothetical proteins) is given in brackets.

c Strain SAG 263-11, initially described as P. wickerhamii.

d A. protothecoides mitochondrion includes also two pseudogenes.

e P. wickerhamii mitochondrion and plastid include two tRNA-Met genes.

Plastid-based maximum likelihood phylogenomic analysis was performed using IQ-TREE v1.6.12 (Nguyen et al., 2015; Chernomor et al., 2016) with 1,000 bootstrap replicates on all 79 nonhypothetical, protein-coding genes present in the plastid genomes of the 24 investigated taxa. All genes were translated into amino acid sequences, aligned with MAFFT v7.271 (Katoh and Standley, 2013), and concatenated in Geneious 10.2.6 (Kearse et al., 2012) to produce a single alignment with total length of 37,109 amino acids, which was subsequently used as the input dataset for tree reconstruction. The sequence evolution model was selected automatically by IQ-TREE (*-m TEST* parameter; Kalyaanamoorthy et al., 2017) for every partition (gene), and the selected models are shown in **Supplementary Table 1**.

Gene synteny analysis was performed with MAUVE v2.3.1 plugin (Darling et al., 2010), integrated into Geneious 10.2.6 software (Kearse et al., 2012).

The mitochondrial and plastid genomes of *P. wickerhamii* were deposited in the GenBank under MN794237 and MN794236 accession numbers, respectively.

All raw sequence data produced in this study were deposited in the NCBI Short Reads Archive (SRA) under project numbers PRJNA646401 (mitochondrion genome) and PRJNA646400 (plastid genome).

## Results and Discussion

**Figure 1:**
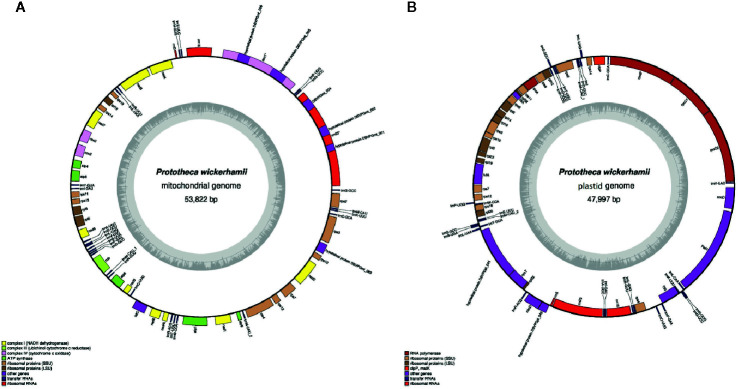
P. *wickerhamii* mitochondrion **(A)** and plastid **(B)** circular plot. Genes (filled boxes) located outside/inside the map are transcribed clockwise/counterclockwise. tRNA genes are indicated by the “trn” followed by one-letter amino acid code and anticodon given behind the dash. Innermost circle represents GC content.

The *P. wickerhamii* mitochondrial DNA (mtDNA), comprising 53,878 and 3,861 reads for Illumina and PacBio, respectively and a 475-fold coverage, was AT-rich, circular mapping molecule (**Figure 1A**, **Table 1**). It was characterized with 42.4% of noncoding DNA including introns (36.1% excluding introns) and an average intergenic space of 340.6 bp. Overall, the *P. wickerhamii* mtDNA architecture mirrored *P. xanthoriae* mtDNA (Wolff et al., 1994; Severgnini et al., 2018; Jagielski et al., 2019). The *P. wickerhamii* mitochondrial genome was significantly larger than that of *P. ciferrii* and *P. bovis* (**Table 1**). These differences might be explained by putative rearrangements and/or reduction events. Exemplarily, in *P. wickerhamii* and *P. xanthoriae*, the *cox1* gene contains three (4,975 bp) and four exons (5,376 bp), respectively, whereas in *P. bovis* and *P. ciferrii*, it is a single exon gene of 1,574 bp. Noteworthy, in the former two species, the *cox1* gene contains two additional genes (in *P. wickerhamii* designated as *DBVPGmt_008 and DBVPGmt_009*), which are missing in the mitochondrial genomes of *P. bovis* and *P. ciferrii* (**Figure 1A**).

The mitochondrion of *P. wickerhamii* encoded 38 proteins, a number that almost equaled that of *A. protothecoides* (39), yet being higher than in other analyzed species, where it ranged from 32 (*C. variabilis*) to 37 (*Helicosporidium* sp.) (**Table 1**).

Mitochondria and plastids originated from a primary endosymbiotic event, yet the subsequent evolution of the two organelles differ. Whereas mitochondria have evolved in a vertical inheritance, plastid evolution has involved both vertical and horizontal spread (Archibald, 2015; Martin et al., 2015; de Vries and Archibald, 2018).

A standard set of 32 mitochondrial protein-coding genes was present in *P. wickerhamii*, namely ribosomal proteins, apocytochrome b, subunits of the ATPase, cytochrome oxidase, NADH dehydrogenase complexes, and Twin-arginine translocation protein. Almost all of them were found among the analyzed species (**Supplementary Table 2**). One exception was *atp8* coding for ATP synthase F0 subunit 8, which was demonstrated in all algal species but *P. xanthoriae* (**Supplementary Table 2**). Furthermore, the *rpl10* gene coding for a ribosomal protein was found only in *P. ciferrii* and *P. bovis*. Transfer of the *atp8* gene from the mitochondrial genome to the nuclear genome had already been reported in various eukaryotic lineages, including ciliates (Burger et al., 2000), apicomplexans, dinoflagellates (Slamovits et al., 2007), and Chlorophyceae (Martínez-Alberola et al., 2019). The *rpl10* gene was lost several times in Chlorophytes, including Chlorophyceae, Ulvophyceae, perhaps Prasinophyceae, and some Trebouxiophyceae (Martínez-Alberola et al., 2019). Whether the *atp8* and *rpl10* were lost entirely or transferred to the protothecal nuclear genomes remains to be answered. Overall, the mitochondrial gene content appears to be highly conserved among the analyzed species.

A total of five introns in two genes (2/67; 3%) were characterized in *P. wickerhamii* mtDNA, with a total length of 7,016 bp (**Table 1**). Those introns split the *cox1* and *rrn23*, into three and four exons, respectively. A more complex intron structure, with up to seven introns and the total length reaching 8,200 bp was observed in *P. xanthoriae, C. variabilis*, *A. protothecoides*, and *Helicosporidium* sp. Interestingly, *P. bovis* showed only a single intron in the *rrn23* gene (**Table 1**). In *P. wickerhamii*, four introns were annotated either as LAGLIDADG endonuclease (DBVPGmt_004) or had LAGLIDADG motifs (DBVPGmt_001, DBVPGmt_002, DBVPGmt_009), which indicates a putative endonuclease function for the protein (Pombert and Keeling, 2010). LAGLIDADG motifs, commonly found in group I introns (Haugen and Bhattacharya, 2004), had previously been described in *P. bovis* and *P. xanthoriae*, but not in *P. ciferrii* (**Supplementary Table 2**).

The *P. wickerhamii* plastid DNA (ptDNA), comprised 81,088 and 5,740 reads for Illumina and PacBio, respectively, had 730-fold coverage (**Figure 1B**, **Table 1**). It was similar in size to the plastid of *P. stagnora* and almost double in size compared to plastids of *P. ciferrii* and *P. bovis.* Structurally, the *P. wickerhamii* plastid was compact, with about 25% of noncoding DNA including introns (23.9% excluding introns) and an average intergenic space of 171.2 bp.

The *P. wickerhamii* ptDNA was predicted to contain 35 protein-coding genes, which was somewhat lower compared with *P. xanthoriae* and *P. cutis* (40), yet clearly higher as compared with *P. ciferrii* and *P. bovis* (19). More than twice as much proteins as in *P. wickerhamii* were encoded by the plastid genomes of the two photosynthetic species (**Table 1**).

The so far sequenced plastid genomes of colorless *Prototheca* spp. were shown to be highly reduced due to the elimination of photosynthesis-related genes. Moreover, comparative analyses of the ptDNAs revealed that the gene content for plastid functions was highly conserved among these nonphotosynthetic lineages (Severgnini et al., 2018; Suzuki et al., 2018; Maciszewski and Karnkowska, 2019; Zeng et al., 2019;). The plastid genomes of *Prototheca* spp. lacked cytochrome complex, photosystem I and II proteins, and genes involved in chlorophyll biosynthesis when compared with their photosynthetic relatives, *i.e.* *A. protothecoides* and *C. variabilis.* The gene content differed also between the *Prototheca* species (**Supplementary Table 3**). Only *P. wickerhamii* and *P. xanthoriae* retained all large ribosomal subunits. Only *P. wickerhamii*, *P. xanthoriae*, and *P. cutis* preserved all small ribosomal subunits, two protein quality control genes—*ftsH* and *clpP*, two translation mediating genes—*tufA* and *infA*, and cell division gene—*minD*. Algae that contain *minD* in their plastid genome are exclusively monoplastidic (de Vries and Gould, 2018). However, the number of plastids in *P. wickerhamii* remains unknown (Nadakavukaren and McCracken, 1977; Kiyohara et al., 2006). *P. wickerhamii*, *P. xanthoriae, P. cutis*, and *P. stagnora* retained also all *rpo* subunits, which are absent from the plastid genomes of *P. ciferrii* and *P. bovis* (**Supplementary Table 3**).

As it was previously hypothesized, changes in *ycf1* occur concomitantly with changes in *FtsH* (de Vries et al., 2017). Unexpectedly, *P. wickerhamii* did encode *FtsH* but not *ycf1* (**Supplementary Table 3**). Although *FtsH* is a plastid-encoded component of the photosystem II maintenance machinery, *ycf1* function is still debatable.

Interestingly, *P. wickerhamii*, *P. stagnora*, *P. bovis*, and *P. ciferrii*, in contrast to *P. xanthoriae* and *P. cutis*, lacked six genes for the ATP synthase subunits, typically involved in the photosynthesis (**Supplementary Table 3**). Genes of the ATP synthase/hydrolysis complex were also detected in nonphotosynthetic plastids of a diatom *Nitzschia* despite the lack of genes for photosynthesis, carbon fixation, and chlorophyll production. It has been hypothesized that ATP synthase subunits present in *Nitzschia* may produce a proton gradient between the thylakoids and stroma, which is involved in protein translocation (Kamikawa et al., 2015). Moreover, reconstruction of plastid metabolism of this diatom suggested that the ATP synthase complex might function to regulate activities of plastid proteins involved in amino acid biosynthesis, reductive pentose phosphate pathway, and glycolysis/gluconeogenesis by pumping protons between the stroma and thylakoid lumen (Kamikawa et al., 2017). The function of the ATP synthase in *Prototheca* spp. might be similar. However, it appeared not indispensable in this lineage since most of the known *Prototheca* ptDNAs completely lacked all genes required for the plastid ATP synthase. In the absence of ATP synthase, some ATP might be imported from the cytosol by plastid ATP transporters as it was shown in diatoms (Ast et al., 2009). Still, whether or not that is the case in *Prototheca* cannot be answered without plastid proteome reconstruction. At this place, it is worth to note that *P. wickerhamii* was demonstrated, upon electron microscopy studies, to contain double-membraned plastids with starch grains and rudimentary lamellar-like structures (Nadakavukaren and McCracken, 1977; Kiyohara et al., 2006).

The observed differences in the gene content among *Prototheca* spp. may reflect an independent loss of photosynthesis in several protothecal lineages. Therefore, various lineages and species might be at a different stage of the reductive evolution of plastid functions after the loss of photosynthesis.

**Figure 2:**
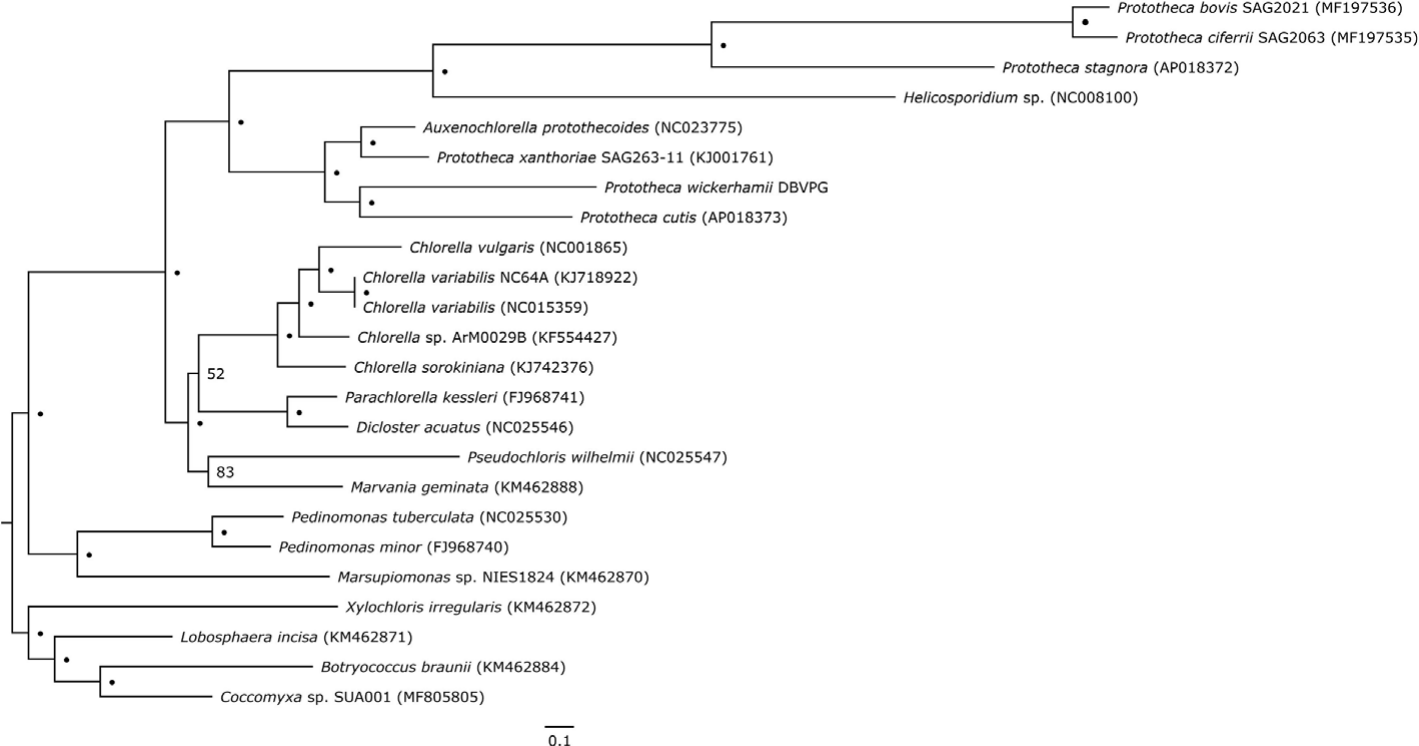
Plastid genome-based phylogenetic tree of *Prototheca* spp. and related genera. Dots (•) represent absolute bootstrap support (100); for nodes without absolute support, numerical bootstrap values are provided. Scale bar at the bottom indicates 0.1 substitution per 10 amino acid positions.

Plastid genome-based phylogeny resolved *Prototheca* as a polyphyletic genus, composed of two major clades (**Figure 2**). The first clade contains two pairs of sister species: *P. cutis* and *P. wickerhamii*, and *P. xanthoriae* and *Auxenochlorella protothecoides*. The second clade contains *P. stagnora*, *P. bovis*, and *P. ciferrii*, with *Helicosporidium* sp. situated on a long branch at its base. All nodes within the aforementioned clades have absolute bootstrap support. Despite limited taxon sampling, our results are fully concordant with *cytb*-based single-gene phylogeny of *Prototheca* and their relatives by Jagielski et al., 2019. Although no plastid genome sequence is available for the *Prototheca* lectotype strain, which is *P. zopfii* ATCC 16533, the recent work of Jagielski et al., 2019 resolves the phylogenetic position of this species as sister to *P. ciferrii*. Therefore, the lectotype would certainly be within the second of the aforementioned clades in our phylogeny, suggesting that only this clade should be recognized as the genus *Prototheca.* Except *A. protothecoides*, the rest of the analyzed species, including *Prototheca* spp. and *Helicosporidium* sp., are secondary nonphotosynthetic Trebouxiophyceae. The tree topology suggests that photosynthesis has been lost at least three times in this lineage, first in *P. xanthoriae*, the second time in the common ancestor of *P. wickerhamii* and *P. cutis*, and the third time in the second clade encompassing *Helicosporidium*, *P. stagnora*, *P. bovis*, and *P. ciferrii*. Three independent losses of photosynthesis in this group are in agreement with earlier reconstructions (Suzuki et al., 2018), and are an excellent example of convergent reductive evolution, reflected in the nearly identical plastid-genome complement in all the *Prototheca* species.

**Figure 3:**
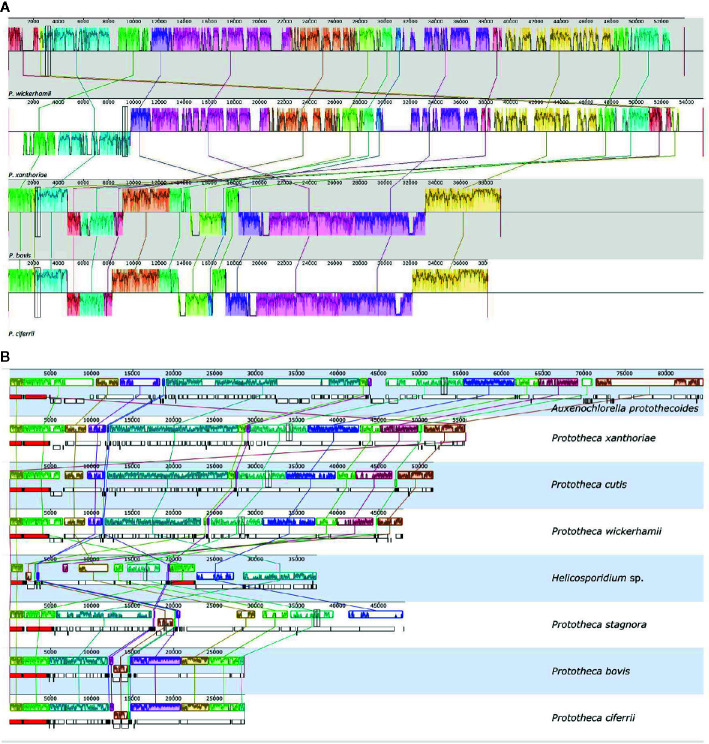
Comparison of the gene order in the mitochondrial **(A)** and plastid **(B)** genomes of the analyzed species. Syntenic regions are marked with matching colors and connected by lines. Numbers indicate nucleotide positions in the mt- and ptDNA.

The mtDNA gene order analysis revealed two lineages among the *Prototheca* spp. investigated: the first allocated *P. wickerhamii* and *P. xanthoriae*, the second contained *P. ciferrii* and *P. bovis* (**Figure 3A**). Not surprisingly, highly syntenic pairs of genomes represented closely related taxa (Jagielski et al., 2019).

The ptDNA protein-coding gene order was exactly identical in the entire clade containing *A. protothecoides, P. wickerhamii, P. cutis*, and *P. xanthoriae*—a sole rearrangement was observed in *P. cutis*, where a small block of three tRNA-coding genes (*trnG, trnH*, and *trnL*) was inverted. An overall similar, yet definitely separate ptDNA synteny type was observed in the other clade of *Prototheca*, composed of *P. stagnora, P. bovis*, and *P. ciferrii* (**Figure 3B**). In this group, four locally collinear ptDNA blocks are translocated in comparison to the first *Prototheca* clade, with a fifth (*tilS-rps4*) block being additionally inverted, which is a unique case of protein-coding gene inversion in protothecan ptDNA since their diversification from the last common ancestor of the entire genus.

The presence of two *Prototheca* lineages, evidenced by pt- and mtDNA structure raises a question, if the human- and cattle-associated clades, represented by *P. wickerhamii* and *P. bovis*, respectively, have acquired pathogenic features independently and in parallel rather than from a common ancestor. The WGS data of *Prototheca* spp. will give a better understanding of the pathobiology and evolution of this genus.

## Conclusions

In conclusion, this study provides a first, brief insight into the organellar genomes of *P. wickerhamii*. The mtDNA of *P. wickerhamii* preserved its functionality similar to other related organisms, with its size extension, mostly due to a higher number of introns (five in both *P. wickerhamii* and *P. xanthoriae*), as well as some unique putative genes unseen in other species (*P. bovis* and *P. ciferrii*). Compact and simplified structure was observed in the *P. wickerhamii* plastid genome, driven by the lack of photosynthesis-related genes. The architecture of the *P. wickerhamii* mitochondrial and plastid genomes resembles more that of closely related saprophytic *P. xanthoriae* than of pathogenic *P. ciferrii* and *P. bovis*.

## Data Availability Statement

The datasets presented in this study can be found in online repositories. The names of the repository/repositories and accession number(s) can be found in the article/supplementary material.

## Author Contributions

ZB performed culturing, analyzed the data, and wrote the article. JG performed DNA isolation and genome sequencing (Illumina). RG provided genome annotation. PS performed synteny analysis. JP performed genome sequencing (PacBio). KM performed phylogenetic analysis, reviewed synteny analysis and genome annotation. AG performed RNA-seq analysis. AK performed phylogenetic analysis and reviewed the manuscript. TJ conceptualized and supervised the study, provided the funding, critical revision. All authors contributed to the article and approved the submitted version.

## Funding

The study was financed by the National Science Centre grants «PRELUDIUM» (2013/09/N/NZ2/00248) and «SONATA» (2014/15/D/NZ7/01797). Additionally, AK and KM were supported by the National Science Centre grant (“SONATA” 2016/21/D/NZ8/01288) and EMBO Installation Grant (to AK).

## Conflict of Interest

The authors declare that the research was conducted in the absence of any commercial or financial relationships that could be construed as a potential conflict of interest.

## References

[B1] Archibald J. M. (2015). Endosymbiosis and eukaryotic cell evolution. Curr. Biol. 25, 911–921. 10.1016/j.cub.2015.07.055 26439354

[B2] Ast M. Gruber A. Schmitz-Esser A. Neuhaus H. E. Kroth P. G. Horn M. (2009). Diatom plastids depend on nucleotide import from the cytosol. PNAS 106, 3621–3626. 10.1073/pnas.0808862106 19221027 PMC2642474

[B3] Burger G. Zhu Y. Littlejohn T. G. Greenwood S. J. Schnare M. N. Lang B. F. (2000). Complete sequence of the mitochondrial genome of *Tetrahymena pyriformis* and comparison with *Paramecium aurelia* mitochondrial DNA. J. Mol. Biol. 297, 365–380. 10.1006/jmbi.2000.3529 10715207

[B4] Carver T. Harris S. R. Berriman M. Parkhill J. McQuillan J. A. (2012). Artemis: an integrated platform for visualization and analysis of high-throughput sequence-based experimental data. Bioinformatics 28, 464–469. 10.1093/bioinformatics/btr703 22199388 PMC3278759

[B5] Chan P. P. Lowe T. M. (2019). tRNAscan-SE: searching for tRNA genes in genomic sequences. Methods Mol. Biol. 1962, 1–14. 10.1007/978-1-4939-9173-0_1 31020551 PMC6768409

[B6] Chernomor O. von Haeseler A. Minh B. Q. (2016). Terrace aware data structure for phylogenomic inference from supermatrices. Syst. Biol. 65, 997–1008. 10.1093/sysbio/syw037 27121966 PMC5066062

[B7] de Vries J. Gould S. B. (2018). The monoplastidic bottleneck in algae and plant evolution. J. Cell Sci. 13, jcs203414. 10.1242/jcs.203414 28893840

[B8] Darling A. E. Mau B. Perna N. T. (2010). progressiveMauve: multiple genome alignment with gene gain, loss and rearrangement. PLoS One 5, e11147. 10.1371/journal.pone.0011147 20593022 PMC2892488

[B9] de Vries J. Archibald J. M. (2018). Plastid genomes. Curr. Biol. 28, 336–337. 10.1016/j.cub.2018.01.027 29689202

[B10] de Vries J. Archibald J. M. Gould S. B. (2017). The carboxy terminus of *ycf1* contains a motif conserved throughout >500 myr of streptophyte evolution. Genome Biol. Evol. 9, 473–479. 10.1093/gbe/evx013 28164224 PMC5381667

[B11] Haugen P. Bhattacharya D. (2004). The spread of LAGLIDADG homing endonuclease genes in rDNA. Nucleic Acids Res. 32, 2049–2057. 10.1093/nar/gkh520 15069127 PMC390371

[B12] Jagielski T. Lagneau P. E. (2007). Protothecosis. A pseudofungal infection. J. Mycol. Méd 17, 261–270. 10.1016/j.mycmed.2007.08.003

[B13] Jagielski T. Gawor J. Bakuła Z. Zuchniewicz K. Gromadka R. (2017). Żak I An optimized method for high quality DNA extraction from microalga *Prototheca wickerhamii* for genome sequencing. Plant Methods 13, 105. 10.1186/s13007-017-0228-9 PMC5627410 29026433

[B14] Jagielski T. Bakula Z. Gawor J. Maciszewski K. Dyląg M. Nowakowska J. (2019). The genus *Prototheca* (Trebouxiophyceae, Chlorophyta) revisited: implications from molecular taxonomic studies. Alg. Res. 43, 101639. 10.1016/j.algal.2019.101639

[B15] Kalyaanamoorthy S. Minh B. Q. Wong T. K. F. von Haeseler A. Jermiin L. S. (2017). ModelFinder: fast model selection for accurate phylogenetic estimates. Nat. Methods 14, 587–589. 10.1038/nmeth.4285 28481363 PMC5453245

[B16] Kamikawa R. Tanifuji G. Ishikawa S. A. Ishii K. Matsuno Y. Onodera N. T. (2015). Proposal of a twin arginine translocator system-mediated constraint against loss of ATP synthase genes from nonphotosynthetic plastid genomes. Mol. Biol. Evol. 32, 2598–2604. 10.1093/molbev/msv134 26048548

[B17] Kamikawa R. Moog D. Zauner S. Tanifuji G. Ishida K. I. Miyashita H. (2017). A non-photosynthetic diatom reveals early steps of reductive evolution in plastids. Mol. Biol. Evol. 34, 2355–2366. 10.1093/molbev/msx172 28549159

[B18] Katoh K. Standley D. M. (2013). MAFFT Multiple Sequence Alignment Software Version 7: Improvements in Performance and Usability. Mol. Biol. Evol. 30, 772–780. 10.1093/molbev/mst010 23329690 PMC3603318

[B19] Kearse M. Moir R. Wilson A. Stones-Havas S. Cheung M. Sturrock S. (2012). Geneious Basic: An integrated and extendable desktop software platform for the organization and analysis of sequence data. Bioinformatics 28, 1647–1649. 10.1093/bioinformatics/bts199 22543367 PMC3371832

[B20] Kiyohara N. Osafune T. Ehara T. Hiroshi H. Ito-Kuwa S. Aoki S. (2006). Immuno-electron microscopic studies on plastid DNA and photosynthetic proteins in *Prototheca wickerhamii* . Cytol. - CYTOL. TOKYO 71, 309–314. 10.1508/cytologia.71.309

[B21] Lass-Flörl C. Mayr A. (2007). Human protothecosis. Clin. Microbiol. Rev. 20, 230–242. 10.1128/CMR.00032-06 17428884 PMC1865593

[B22] Maciszewski K. Karnkowska A. (2019). Should I stay or should I go?Retention and loss of components in vestigial endosymbiotic organelles. Curr. Opin. Genet. Dev. 58-59, 33–39. 10.1016/j.gde.2019.07.013 31466038

[B23] Martin W. F. Garg S. Zimorski V. (2015). Endosymbiotic theories for eukaryote origin. Phil. Trans. R Soc. B 370, 20140330. 10.1098/rstb.2014.0330 26323761 PMC4571569

[B24] Martin M. (2011). Cutadapt removes adapter sequences from high-throughput sequencing reads. EmbNET J. 17, 10–12. 10.14806/ej.17.1.200

[B25] Martínez-Alberola F. Barreno E. Casano L. M. Gasulla F. Molins A. del Campo E. M. (2019). Dynamic evolution of mitochondrial genomes in Trebouxiophyceae, including the first completely assembled mtDNA from a lichen-symbiont microalga (Trebouxia sp. TR9). Sci. Rep. 9, 8209. 10.1038/s41598-019-44700-7 31160653 PMC6547736

[B26] Nadakavukaren M. J. McCracken D. A. (1977). An ultrastructural survey of the genus *Prototheca* with special reference to plastids. Mycopathologia 61, 117–119. 10.1007/BF00443840 27542819

[B27] Nguyen L. T. Schmidt H. A. von Haeseler A. Minh B. Q. (2015). IQ-TREE: A fast and effective stochastic algorithm for estimating maximum likelihood phylogenies. Mol. Biol. Evol. 32, 268–274. 10.1093/molbev/msu300 25371430 PMC4271533

[B28] Pearson W. R. Wood T. Zhang Z. Miller W. (1997). Comparison of DNA sequences with protein sequences. Genomics 46, 24–36. 10.1006/geno.1997.4995 9403055

[B29] Pombert J. F. Keeling P. J. (2010). The mitochondrial genome of the entomoparasitic green alga *Helicosporidium* . PLoS One 5, e8954. 10.1371/journal.pone.0008954 20126458 PMC2813288

[B30] Ruan J. Heng L. (2020). Fast and accurate long-read assembly with wtdbg2. Nat. Methods, 17, 155–158. 31819265 10.1038/s41592-019-0669-3 PMC7004874

[B31] Severgnini M. Lazzari B. Capra E. Chessa S. Luini M. Bordoni R. (2018). Genome sequencing of *Prototheca zopfii* genotypes 1 and 2 provides evidence of a severe reduction in organellar genomes. Sci. Rep. 8, 14637. 10.1038/s41598-018-32992-0 30279542 PMC6168571

[B32] Slamovits C. H. Saldarriaga J. F. Larocque A. Keeling P. J. (2007). The highly reduced and fragmented mitochondrial genome of the early-branching dinoflagellate *Oxyrrhis marina* shares characteristics with both apicomplexan and dinoflagellate mitochondrial genomes. J. Mol. Biol. 372, 356–368. 10.1016/j.jmb.2007.06.085 17655860

[B33] Suzuki S. Endoh R. Manabe R. I. Ohkuma M. Hirakawa Y. (2018). Multiple losses of photosynthesis and convergent reductive genome evolution in the colourless green algae *Prototheca* . Sci. Rep. 17, 940. 10.1038/s41598-017-18378-8 PMC5772498 29343788

[B34] Tillich M. Lehwark P. Pellizzer T. Ulbricht-Jones E. S. Fischer A. Bock R. (2017). GeSeq – versatile and accurate annotation of organelle genomes. Nucleic Acids Res. 45, 6–11. 10.1093/nar/gkx391 PMC5570176 28486635

[B35] Todd J. R. Matsumoto T. Ueno R. Murugaiyan J. Britten A. King J. W. (2018). Medical phycology 2017. Med. Mycol. 56, 188–204. 10.1093/mmy/myx162 29767780

[B36] Walker B. J. Abeel T. Shea T. Priest M. Abouelliel A. Sakthikumar S. (2014). Pilon: an integrated tool for comprehensive microbial variant detection and genome assembly improvement. PLoS One 9, e112963. 10.1371/journal.pone.0112963 25409509 PMC4237348

[B37] Wolff G. Kück U. (1996). Transcript mapping and processing of mitochondrial RNA in the chlorophyte alga Prototheca wickerhamii. Plant Mol. Biol. 30, 577–595. 10.1007/BF00049333 8605307

[B38] Wolff G. Plante I. Lang B. F. Kück U. Burger G. (1994). Complete sequence of the mitochondrial DNA of the chlorophyte alga *Prototheca wickerhamii.* Gene content and genome organization. J. Mol. Biol. 18, 75–86. 10.1006/jmbi.1994.1210 8133522

[B39] Yan D. Wang Y. Murakami T. Shen Y. Gong J. Jiang H. (2015). *Auxenochlorella protothecoides* and *Prototheca wickerhamii* plastid genome sequences give insight into the origins of non-photosynthetic algae. Sci. Rep. 25, 14465. 10.1038/srep14465 PMC4585924 26403826

[B40] Zeng X. Kudinha T. Kong F. Zhang Q. Q. (2019). Comparative genome and transcriptome study of the gene expression difference between pathogenic and environmental strains of *Prototheca zopfii* . Front. Microbiol. 7, 443. 10.3389/fmicb.2019.00443 PMC6416184 30899253

